# Correlation of Electrically Evoked Compound Action Potential Amplitude Growth Function Slope and Anamnestic Parameters in Cochlear Implant Patients—Identification of Predictors for the Neuronal Health Status

**DOI:** 10.3390/life11030203

**Published:** 2021-03-05

**Authors:** Lutz Gärtner, Katharina Klötzer, Thomas Lenarz, Verena Scheper

**Affiliations:** 1 Department of Otolaryngology, Hannover Medical School, 30625 Hannover, Germany; gaertner.lutz@mh-hannover.de (L.G.); katharina.kloetzer@web.de (K.K.); Lenarz.Thomas@mh-hannover.de (T.L.); 2 Cluster of Excellence “Hearing4All”, 30625 Hannover, Germany

**Keywords:** ECAP AGF slope, prognostic factors, neuronal function, spiral ganglion neurons

## Abstract

Cochlear implants (CI) are the treatment of choice in profoundly deaf patients. Measuring the electrically evoked compound action potential (ECAP) has become an important tool for verifying the function of the spiral ganglion neurons (SGN), which are the target cells of the CI stimulation. ECAP measurement is only possible after electrode insertion. No information about the neuronal health status is available before cochlear implantation. We investigated possible correlations between the ECAP amplitude growth function (AGF) slope and anamnestic parameters to identify possible predictors for SGN health status and therefore for CI outcome. The study included patients being implanted with various electrode array lengths. Correlation analysis was performed for the mean AGF slope of the whole array, for separate electrodes as well as for grouped electrodes of the apical, medial, and basal region, with duration of deafness, age at implantation, residual hearing (grouped for electrode length), and etiology. The mean ECAP AGF slopes decreased from apical to basal. They were not correlated to the length of the electrode array or any etiology. For the mean of the full array or when grouped for the apical, middle, and basal part, the ECAP AGF slope was negatively correlated to the duration of hearing loss and the age at implantation. Since a significant negative correlation of the ECAP AGF slope and age at cochlear implantation and duration of deafness was observed, this study supports the statement that early implantation of a CI is recommended for sensorineural hearing loss. Additional factors such as the cochlear coverage and insertion angle influence the ECAP AGF slope and performance of the patient and should be included in future multifactorial analysis to study predictive parameters for the CI outcome.

## 1. Introduction

**Figure 1:**
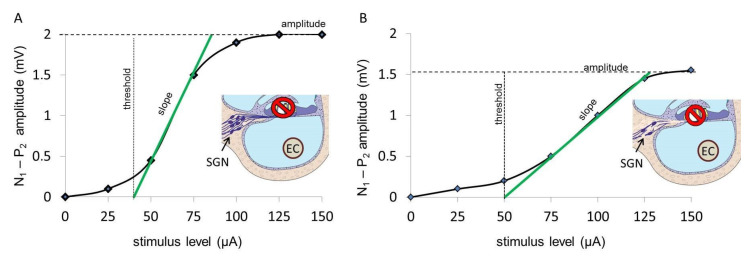
Graphical illustration of ECAP AGF slopes in individuals with more vital SGN (**A**), where the slope is steeper, and for less healthy SGN (**B**), where the slope is more flattened. The differences between the negative (N) and positive (P) peaks N1–P2 for the various stimulation levels (µA) are plotted. The slope of the growth function is marked in green. The drawn inlets in A and B illustrate the scala tympani and organ of Corti with erased hair cells and the surviving spiral ganglion neurons (SGN) with a higher number as seen in individuals with a more steep AGF slope (**A**) and a reduced number of SGN, resulting in a more shallow slope and a reduced maximum ECAP amplitude (**B**), respectively. An electrode contact (EC) of the CI array located in the scaly tympani is shown as the source of the electrical stimulation.

Worldwide, 466 million people suffer from disabling hearing loss with negative effects in social, emotional, and economic capacity due to a limited ability of communication [1]. Patients with severe to profound sensorineural hearing loss are usually candidates to receive a cochlear implant (CI). The CI’s electrode array is implanted into the scala tympani of the cochlea for electrical stimulation of the spiral ganglion neurons (SGN), which subsequently leads to sound perception (Figure 1). The benefits patients may get from a CI vary widely and are today not predictable. There are very promising studies suggesting preoperative speech audiometric parameters as a predictor for the minimum speech perception obtained with CI [2]. Other approaches focus on predicting post-operative outcomes using genetic factors [3]. Those methods can only make predictions within certain patient groups. At least, the presence of a healthy and sufficiently large population of SGN that is able to transduce the encoded auditory information via the afferent auditory system to the auditory cortex has to be considered as prerequisite for cochlear implant outcome. The function and number of SGN correlates, at least in rodents, with the slopes of the electrically evoked compound action potential (ECAP) of the auditory nerve [4,5]. ECAP measurements in human CI users were also indicating such correlation [6,7,8,9]. Steeper slopes of ECAP input/output functions have been found to be generally associated with higher SGN density [10].

In contrast to animal models, histological information on SGN number and electrophysiological data in humans are rare. It is known that, as in animal models [11,12], in hearing impaired patients, the SGN degenerate over time [13,14]. A statement about the electro-responsivity, and thus the health, of the cells could not be made because of the histological evaluation only. It may be that although the cell count remains stable after deafening over the years, the function of the neurons, and thus their electrical responsiveness, decreases. A high number of stimulated neurons is considered to positively influence the outcome of the CI in patients [15,16,17,18]. However, a relation between the number of surviving SGN and CI performances has not been found yet [19,20], since the correlation of histology and electrophysiology in humans is only possible postmortem. Instead, the electrically evoked compound action potential (ECAP) measurement can be used to indirectly describe the number and function of the SGN, giving some prognostic information about the benefit one may receive by cochlear implantation. To perform the ECAP measurement, an inserted electrode array inside the cochlea is necessary. To predict the postoperative performance with a CI, it would be desirable to have an indirect measure of the functional status of the auditory nerve. For this purpose, we investigated the correlation of the ECAP amplitude growth function (AGF) slope with the anamnestic parameters duration of deafness, age at implantation, etiology, and residual hearing.

## 2. Materials and Methods

### 2.1. ECAP AGF Slope

In CI users, it became possible to record the ECAP via the inserted electrode array [21], and this procedure has been implemented into the clinical software of all CI manufactures. Due to its short latency, the P1 wave cannot be recorded, and the ECAP response consists of an N–P complex, which corresponds to the N1–P2 complex of the EABR recording. The ECAP amplitude is defined as the difference between the electric potential of these characteristic negative (N) and positive (P) peaks [22]. With increasing stimulation level, the ECAP amplitude is rising according to a sigmoid-shaped amplitude growth function (AGF; Figure 1). A higher slope value (mV/µA) is expected to correlate with healthier SGN, whereas lower slope values are expected to be found at lower SGN densities.

### 2.2. ECAP Measurement

At our clinic, ECAP measurements are routinely performed intra- as well as postoperatively to receive information on the electrode–nerve interface. In this retrospective analysis, the postop measurements are used, which were recorded via the clinical software MAESTRO (MED-EL, Austria) using the ART (Auditory Nerve Response Telemetry) task. Within the ART task, “Alternating Polarity” is used as artifact reduction paradigm per default. Two stimuli are applied consecutively, one with the anodic leading phase and another with the cathodic leading phase. By averaging the responses of these two pulses, the artifact can be reduced [22]. In addition, the zero amplitude template subtraction algorithm, implemented in the ART task, was applied to all recordings to compensate for the amplifier artifact. Thereby, also a linear drift artifact will be removed automatically through rectification.

Recording parameters like number of steps, number of averages, minimum and maximum stimulation level, and pulse duration were chosen differently depending on the aim of the measurement. To investigate the electrode–nerve interface, a few measurement points are sufficient. To calculate an ECAP threshold, more points are necessary to establish a regression line through the linear region of the AGF. Each recording was reviewed with respect to ECAP responses. The linear regression line, automatically generated by the clinical software MAESTRO, was corrected to fit data points within the linear region, as shown in the examples of Figure 1. From these regression lines, ECAP threshold and slope of the AGF were calculated; 219 patients fulfilled these criteria. Those patients were checked for complete documentation of the anamnestic parameters, resulting in 139 patients being included.

In MED-EL CI implant devices, electrode array lengths are 16, 20, 24, 28, and 31.5 mm. All arrays were inserted to their full length. Current level and charge level are approximately equal to SI units: 1 cu ≈ 1 µA, 1 qu ≈ 1 nC.

Since the electrode arrays consisted of 12 electrode contacts, the postoperative ECAP measurement dataset can have a maximum of 12 measurements. There are patients where all 12 electrodes were stimulated successfully. However, there are also patients in whom the ECAP AGF slope of some electrodes is documented as not measurable (e.g., it is too loud for the patient, or an exact value cannot be determined because too few data points are available to calculate the slope) or not available (which means no measurement was made on this electrode for unknown reasons). Those missing values were either very conservatively replaced by single imputation of the worst possible value 0 (analysis A), or excluded from analysis (analysis B) [23]. ECAP slope was shown to increase from the basal towards the apical region [8]. To analyze effects correlating with the location of the electrode contact, different groups of electrodes were defined over which the averaged ECAP slope was calculated:

1. Whole array: electrodes 1–12
2. Apical region: electrodes 1–3
3. Medial region: electrodes 4–9
4. Basal region: electrodes 10–12
5. Single apical electrode no. 1
6. Single apical electrode no. 2
7. Single apical electrode no. 3

### 2.3. Anamnestic Parameters

This retrospective study investigated whether the ECAP AGF slope, and therefore presumably the function of SGN, correlates with data that are available before cochlear implantation: the duration of deafness, the age at implantation, the etiology, or the residual hearing. For all variables, the data from 139 patients were analyzed.

All necessary data were selected from the patient medical files and the CI database at Hannover Medical School and supplemented with the available measurements. The influencing factors were determined as follows:

The duration of deafness is expressed in years and measured from the time of deafness to the date of surgery. Patients deaf for less than one year were set to 0.5 years. All remaining time periods were rounded down to half year steps. There were patients for whom no exact date of deafness was given, because they could not give or did not know the exact date of deafness, or because they had some residual hearing before surgery and were therefore not considered deaf. In these cases, the date when the patient was considered hard of hearing was used for calculation.

Age at implantation is indicated in years. The period from birth to implantation is given. Rounding up and down was the same as for the duration of deafness.

In some cases, the age at implantation can be determined more exactly than the duration of deafness, since the latter is often a subjective parameter and should therefore be considered less strongly when judging on anamnestic parameters.

The influencing factor etiology was classified regarding the occurrence:

1. unknown (N = 39)
2. not documented (N = 39)
3. acute (N = 50)
4. progressive (N = 11)

and regarding the cause of the hearing loss:

1. unknown (N = 7)
2. not documented (N = 40)
3. sudden idiopathic hearing loss, acute hearing loss (N = 42)
4. infection (N = 12)
5. syndrome, hereditary (N = 15)
6. trauma, ototoxins (N = 23)
7. presbyacusis (N = 0)

**Figure 2:**
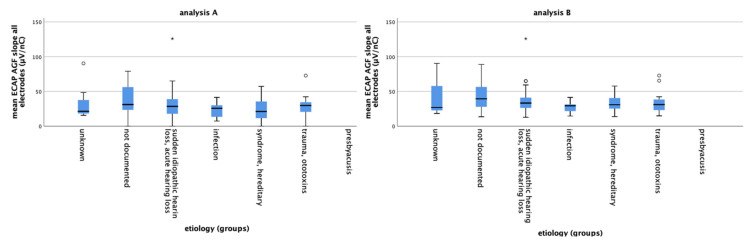
The mean ECAP AGF slopes of the different etiology groups for data set **A** (**left**) and data set **B** (**right**) are plotted. The circles and asterisk indicate individual points that are outliers with small circles indicating “out” values and stars indicating “far out” values.

The etiology and mean ECAP AFG slope of all electrodes were plotted (Figure 2) to decide for the correct procedure for statistical analysis. “Unknown” was defined as reference category so that the significance of all other groups has to be considered on the basis of this group. As no patient in our study group suffered from presbyacusis, this cause was not taken into account for further analysis.

To determine the residual hearing, all available pure tone audiograms of each individual patient were examined, and the audiogram, which was created as close as possible to the date of the first fitting, was taken. The frequencies 125, 250, 500, and 1000 Hz were evaluated for the respective hearing loss (dB SPL), whereas the higher frequencies (2, 4, 8 kHz) were not taken into account, since hearing was lost in these frequency regions; 750 Hz was not included into the analysis, since not every audiogram included this measurement. To detect the frequency-specific acoustic hearing loss, air conduction hearing loss was measured using a calibrated audiometer according to DIN EN 60318 as previously described in detail [24].

Analysis: SPSS (IBM, Armonk, NY, USA) and GraphPad Prism (version 5.0 for Windows, GraphPad Software, San Diego, CA, USA) were used for analysis. Data were checked for normal distribution using D’Agostino–Pearson Test, and ECAP AGF slopes of grouped and single electrodes were compared using Mann–Whitney test. Pearson correlation was subsequently applied. A model was created in which univariate linear regressions were performed followed by examination of all significant variables in a common model by multivariate analysis. The analysis was performed with the data set of missing values being set to zero (analysis A) as well as with a data set where missing values were excluded (analysis B).

Boxplots show the median, quartiles, and extreme values. The box represents the interquartile (IQ) range, which contains the middle 50% of the records. The whiskers are lines that extend from the upper and lower edge of the box to the highest and lowest values, which are not greater than 1.5 times the IQ range. The line across the box indicates the median. Outliers are cases with values between 1.5 and 3 times the IQ range, i.e., beyond the whiskers and marked with a small circle. Extremes are cases with values more than three times the IQ range and marked with a star.

In the univariate analysis, the ECAP AGF slope was defined as the dependent variable and the respective influencing factor as an independent variable. Each influencing factor was examined individually in a regression model to the significance level of 5%. Pearsons squared correlation coefficient *r^2^* was calculated. The factors duration of deafness, age at implantation, etiology grouped by occurrence, and etiology grouped by cause were correlated with the mean ECAP AGF slope values of electrodes covering different regions of the cochlea.

To describe the differences between patients with residual hearing in the low-frequency (apical) region and patients without any residual hearing, ECAP AGF slopes of the apical electrodes 1–3 were included into the analysis. The mean ECAP AGF slope of electrodes 1–3 was correlated with the hearing loss values of the frequencies 125, 250, 500, and 1000 Hz. Additionally, the frequency specific hearing loss was correlated with each single electrodes (1, 2, and 3) ECAP AGF slope.

All linear regressions of the residual hearing were made separately for each electrode array length. Since there are only two patients for the 16 mm implant model, it was omitted. Finally, a multivariate regression was used to determine whether all significant analyses correlate.

## 3. Results

### 3.1. ECAP AGF Slope

**Figure 3:**
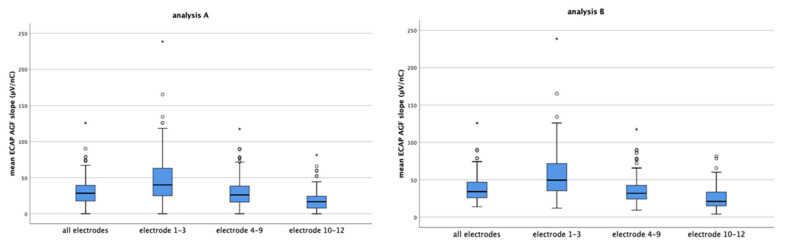
The mean ECAP AGF slopes of all electrodes and grouped electrodes 1–3, 4–9, and 10–12 for analysis **A** (**left**) and analysis **B** (**right**) are shown. The circles and asterisk indicate individual points that are outliers.

The mean ECAP AGF slope of all patients electrodes for analysis A = missing values set to 0 and analysis B = missing values excluded are 31.10 µV/nC and 37.26 µV/nC, respectively. Grouped for electrodes 1–3, 4–9, and 10–12, the mean ECAP AGF slopes are 46.52 µV/nC, 29.77 µV/nC, and 18.32 µV/nC (analysis A, Figure 3, left graph) and 54.73 µV/nC, 34.50 µV/nC and 25.06 µV/nC (analysis B, Figure 3, right graph), respectively. None of the data are normally distributed. Using Mann–Whitney test, significant differences between the ECAP AGF slopes of the different electrode groups are detected. For data set A and B, the mean slopes of electrode 1–3 differ significantly from the basal and medial electrodes’ mean slopes (*p* < 0.001 for both pairings). Additionally, the slopes measured on the basal electrodes were significantly reduced compared to the medial electrodes (*p* < 0.001).

**Figure 4:**
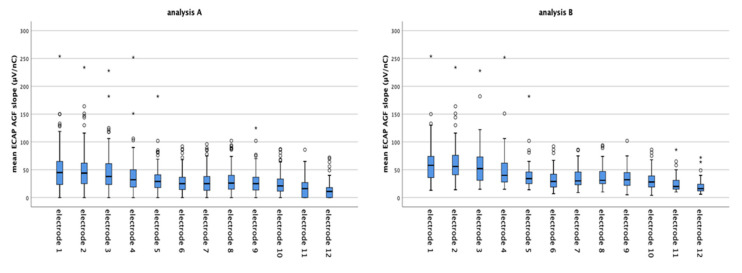
The mean ECAP AGF slopes of the separate electrodes decreases continuously from electrode 1 (apical) to electrode 12 (basal) in both data sets analyzed (analysis **A** left graph; analysis **B** right graph). Imputing missing values as in data set A results in lower mean ECAP AGF slopes than excluding missing values from analysis (data set B). The circles and asterisk indicate individual points which are outliers.

The mean ECAP AGF slopes of the separate electrodes decrease from apical to basal with the mean slopes of electrodes 1, 2, and 3 being between 40 to 50 µV/nC (mean values: electrode 1: 47.61 µV/nC, electrode 2: 48.51 µV/nC, electrode 3: 43.43 µV/nC), and electrode 10, 11, and 12 below 25 µV/nC (data reported based on analysis A, Figure 4 left graph). The same continuous decrease in ECAP AGF slope from apical to basal was observed if analysis B = missing values excluded was performed (Figure 4, right graph; mean values electrode 1: 58.05 µV/nC, electrode 2: 57.14 µV/nC, electrode 3: 52.96 µV/nC),) with data set B resulting in higher mean slopes than data set A.

### 3.2. Duration of Deafness

**Figure 5:**
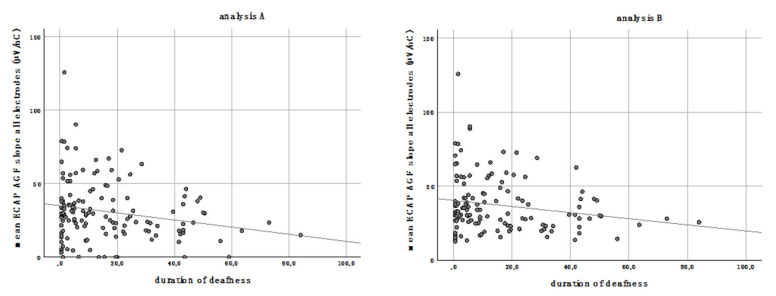
The mean ECAP AGF slope of all electrodes correlates negatively with the duration of deafness in both analyses performed. Analysis **A** (**left graph**): missing values are set to zero; analysis **B** (**right graph**): missing values are excluded.

A significant negative correlation was observed for the duration of deafness and the ECAP AGF slope (Table 1). Patients who were implanted a long time after onset of deafness revealed the shallowest slopes. This correlation was detectable for the mean of all electrodes (*p* = 0.017 for analysis A; *p* = 0.032 for analysis B, Table 1, and Figure 5 (A,B) as well as for the grouped electrodes (Table 1). The correlation of ECAP AGF slopes and grouped electrodes was more pronounced in the basal and middle region compared to the apical region (Table 1).

### 3.3. Age at Implantation

**Figure 6:**
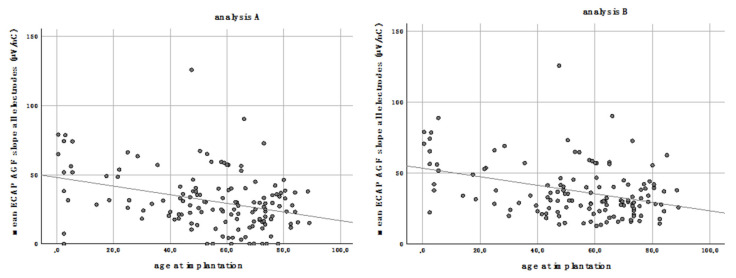
The mean ECAP AGF slope of all electrodes correlates negatively with the age at implantation for analysis **A** (**left**) and **B** (**right**).

The age at implantation did significantly correlate negatively to the mean ECAP AGF slope of all electrodes (analysis A: *p* < 0.001, r^2^ = 0.119; analysis B: *p* < 0.001, r^2^ = 0.134; Figure 6). Patients who received a CI at an advanced age have significantly shallower ECAP AGF slopes than younger patients. This correlation was as well detectable for the grouped electrodes of the basal, middle, and apical part of the array with a more pronounced correlation in the basal and middle region compared to the apical region (Table 1).

### 3.4. Etiology

The evaluation of the individual etiologies is considered in relation to the respective reference category “unknown”. No presbyacusis patient was included into the dataset; therefore, this etiology was excluded from analysis. The evaluation is carried out with the data set of analysis A and B for the mean of all electrodes (Table 2 for data set B), and for electrodes 1–3, 4–9 and 10–12 (Table 3). There is no significant correlation detectable. However, the ECAP AGF slope of the different etiologies can be correlated on the basis of the regression coefficient B. The regression coefficient B indicates by how many units the dependent variable changes when the independent variable changes by one unit, but since the variable “etiology” is a nominal variable, the interpretation here is different. Because “unknown” is the defined reference category, the regression coefficient B indicates by how many units the ECAP AGF slope value of the corresponding category increases or decreases compared to a patient with the etiology “unknown”. In other words: this is reporting the effect estimate, predicting how the slope value may change if the respective etiology is the cause of hearing loss. For example, the ECAP AGF slope of patients with acute hearing loss is predicted to be 5.32 µV/nC smaller than in patients where the etiology is unknown. Infections result in the shallowest, 15.44 µV/nC reduced, slope estimate.

This trend for the effect estimate is pronounced at the apical electrodes 1–3 (Table 3). At these apical electrodes, the prediction for reduced slopes is 27.54 µV/nC (analysis A) and 24.22 µV/nC (analysis B) for infection. For syndromal/hereditary hearing loss, slope reductions of 30.80 µV/nC (analysis A) or 21.84 µV/nC (analysis B) are predicted. Those high effect estimates are not detectable in the middle and basal part of the array (Table 3).

### 3.5. Etiology Grouped by Occurrence of Hearing Loss

The influencing factor etiology was classified regarding the occurrence:

1. Unknown; 2. Not documented; 3. Acute; 4. Progressive.

Again, the analysis was performed using the reference category “unknown” for the mean of all electrodes, electrode 1–3, 4–9, and 10–12. No correlation was detected.

The effect estimates do not show a trend for any of the electrode groups analyses and occurrence of hearing loss (Table 4).

### 3.6. Residual Hearing

To investigate dependencies between residual hearing and ECAP AGF slope the data of data set A and B were analyzed separately for each electrode array length (20, 24, 28, and 31.5 mm). The hearing loss at 125, 250, 500, and 1000 Hz was correlated (Pearson correlation) to the mean slope values of the separate electrodes 1, 2, and 3, and to the mean of electrodes 1, 2, and 3. Only few of these 128 performed analyses have shown significant correlations, which are listed in Table 5.

### 3.7. Multivariate Analysis

In a multivariate analysis of all significant factors in a common model, the factors duration of deafness and age at implantation have a strong negative correlation to the mean ECAP AGF slope of all electrodes, but after multivariate analysis, the age at implantation is the only significant factor affecting the ECAP AGF slope (Table 6).

The multivariate analysis of the mean ECAP AGF slopes of electrodes 1–3 was performed for dataset B but not for data set A, because for the latter, only age at implantation but not duration of deafness is significantly correlated to the respective slopes (Table 1). The multivariate analysis results in a significant correlation for the age at implantation (*p* = 0.036) but not for the duration of deafness (*p* = 0.077).

For electrodes 4–9, the duration of deafness, and the age at implantation, significant negative correlations to the mean ECAP AGF slopes measured were detected (Table 1) and therefore included into the multivariate analysis, which resulted in a significant negative correlation of both variables for both data sets analyzed (A and B) (Table 6).

The mean ECAP AGF slopes of electrodes 10–12 correlated with the duration of deafness and age at implantation (Table 1), and the multivariate analysis resulted in a significant negative correlation of both variables and the respective slopes (Table 6).

### 3.8. Electrode Array Length

The mean ECAP AGF slopes of all electrodes and of the grouped electrodes of the different cochlear implant array lengths used are reported in Table 7. In general, the slopes are higher at the more apical electrodes 1–3 compared to the mean slopes measured at the middle (4–9) or basal part (10–12) of the array.

## 4. Discussion

The purpose of cochlear implantation is to restore functional hearing via an implanted device that electrically stimulates the auditory nerve. Over the last decades, technological advances helped to significantly improve speech perception in implanted patients. Nevertheless there is a large inter-patient variability in the benefit patients have by using their implant. This uncertainty and parameter variability have been shown to affect CI outcomes [25,26,27,28]. Studies investigating factors affecting auditory performance of postlinguistically deaf adults using CI report a negative effect of long duration of severe to profound hearing loss on auditory performance and that CI experience is greater with a steeper learning curve. Patients with longer durations of severe to profound hearing loss are less likely to improve with CI experience than patients with shorter duration of severe to profound hearing loss [28]. Additionally, patient-specific cochlear anatomy has been identified as one factor that determines intracochlear electrode array position [26,29] and a broad range of post-operative speech perception scores [30]. There are ongoing activities to find predictive parameters supporting pre- and perioperative decision making. The outcomes of CI computational models considering parameter uncertainty and variability for the prediction of neural response to support optimization processes for surgical planning and implant design were studied by Mangado and colleagues [31]. Others investigate speech audiometric parameters for their predictive capacity [2]. In contrast to these computational or audiometrical approaches, we aimed to identify predictors based on anamnestic parameters to guide and assist preoperative decisions to optimize the therapy for the individual patient.

From animal models, we know that SGN degenerate over time due to lack of neurotrophic support from the Organ of Corti [32,33]. The functional consequence of a reduced number and decreased function of SGN with ongoing deafness is discussed controversially. For EABRs, excitation thresholds have been reported to become elevated [34,35] or unchanged [36] after deafening [37]. Measurements have shown that the first positive (P1) and negative (N1) wave, the compound action potential (CAP) of the auditory nerve, seems to give reliable information about the SGN health status in animal models [4]. The ECAP amplitude largely depends on factors such as the distance between the stimulation electrode and the excitable tissue, the impedance between the two, and, likewise, on the distance and impedance between the excited neural tissue and the recording electrode [38]. To overcome those unpredictable and confounding factors affecting the amplitude, more recently the ECAP AGF slope was introduced as a more robust measure of the electrode–nerve interface integrity. In animal models, the ECAP AGF slope correlates with the SGN number [37], with the steepest slope occurring in animals with the best nerve survival and the shallowest slope found in animals with a very low nerve survival [10]. We therefore decided to use the AGF slope for our analysis.

We report results of two different approaches on how to deal with missing values. For analysis A, missing slope values were replaced by zero, while for analysis B, missing slope values were excluded. By the conservative statistical approach of setting missing values to zero, averages may be shifted to lower AGF slopes as they are in reality, leading to misinterpretation in terms of judging the slope and therefore the SGN function to be lower than it is. The rationale for this conservative approach is that a missing slope value could indeed refer to a dead region or poor SGN function. On the other hand, spread of excitation usually spans several electrode contacts. If an ECAP response is measurable on one electrode contact, there should be a response on an adjacent contact as well. An exception would represent extracochlear contacts, caused by a migrated electrode array or partial insertion. However, such cases were not within our study group. All patients had auditory perceptions on each of the electrode contacts. ECAP measurements may become unpleasantly loud at higher levels, and this is in clinical routine a reason to abort the measurement. Therefore, analysis B seems to be a more appropriate approach. We report that excluding values results in a small increase of mean ECAP AGF slopes (Figure 1; mean ECAP AGF slope of all patients and all electrodes for analysis A: 31.10 µV/nC; analysis B: 37.26 µV/nC; mean increase: 6.16 µV/nC). When correlating the ECAP AGF slopes to the variables duration of deafness and age at implantation, the differences between the two analyses sets are only small as well. Since the differences are only small, it may be speculated that there is the possibility that the values set to 0 are really lower than the neighboring electrodes suggest. One explanation for this finding may be a selective loss of SGN function with cochlear regions being more affected than others.

Many patients suffering from hearing loss mainly lose their hearing first in the basal, high frequency region of the inner ear and subsequently in the middle and more apical regions. This audiological finding is mirrored in our data where we detect significantly lower slope values at electrodes located in the basal region (electrodes 10–12, mean AGF slope = 18.32 µV/nC), medium values at electrodes positioned in the middle part of the cochlear (electrodes 4–9, mean AGF slope = 29.77 µV/nC), and the highest slopes in the more apical region (electrode 1–3, mean slope = 46.52 µV/nC; all values taken from analysis A, see text above Figure 3). The finding of lower slopes in the basal region and higher slopes at apical electrodes may be correlated with lower (basal) and higher (apical) residual SGN numbers. It can be speculated that regions where the hearing loss lasts longer, i.e., in patients suffering from age related hearing loss, who first lose their high frequency hearing, the SGN number is more reduced than in regions where hearing loss occurred delayed. Such a most severe degeneration of the SGN in the basal half of the cochlear was reported by Zimmermann and colleagues [39], and our electrophysiological data may be a link between the histological finding and the audiological data. Nevertheless, it has to be mentioned that one study did not find differences in SGN density of basal and apical cochlear regions in children ages 10 years and younger [40].

In our dataset, the age at implantation and duration of deafness affect the mean ECAP AGF slope significantly, with age at implantation having the most impact on the slope. If the analysis is focused on the different electrodes of the cochlear implant array, this correlation is still detectable at the apical, middle, and basal region. The final multivariate analysis shows that age at implantation is the most relevant factor influencing the ECAP AGF slope, resulting per year in a 0.28 µV/nC decreased mean AGF slope. Previous studies report that it will be beneficial for patients to be implanted as early in life as possible [28,41]. It was shown that child-implanted patients had larger ECAP amplitudes and steeper AGF linear slopes than adult-implanted patients, suggesting that young CI listeners who were deafened and implanted during childhood may have denser neural populations than older listeners who were deafened and implanted as adults [42]. Our data support this statement and may give an explanation for this by showing that with increasing age, the AGF slope is decreasing. Assuming that the slope depends on the SGN number and function, this suggests that there are fewer and/or less vital SGN in older patients than in younger ones.

The correlation analysis of residual hearing and the ECAP AGF slope did reveal a significant dependency in data set B between electrode 3 of the 24 mm length implant array with the residual hearing at 250 Hz (*p* = 0.041, *r*^2^ = 0.425; Table 5). In data set A, the slope measured on the second electrode contact of 20 mm arrays was significantly correlated to 125, 250, and 500 Hz. Additionally, the mean slopes of electrode 1–3 correlated with the residual hearing threshold at 500 Hz, but in general we did not see a higher AGF slope in patients with better residual hearing (= lower hearing loss), implying that there is no difference in the neuronal function in patients with more residual hearing than in patients with profound hearing loss. However, since the correlated electrodes and frequencies are not matched for the real electrode position in the patient, these results give only a rough estimate about the dependencies of slope value and residual hearing. To perfectly match the data, postoperative images should be analyzed for the electrode position and cochlear coverage, as Mlynski and colleagues state [7]. Those factors should be included in future studies on predictive parameters, since they influence the outcome of cochlear implantation [43,44]. For cochlear coverage, which describes the fraction of the cochlea spiral exposed to electric stimulation after implantation [45], it is shown that the higher the cochlear coverage, the better the hearing performance in terms of speech perception [46] or more natural perception of music quality [43]. This may be due to a higher number of SGN to be stimulated with a higher cochlear coverage, and therefore a possible correlation between cochlear coverage and ECAP AGF slope should be investigated in future studies.

The etiology and duration of deafness are important factors for the estimated outcome in speech perception. Kurz and colleagues present data revealing that an inflammatory disease leading to deafness in combination with a long duration of deafness (10 + years) lead to poorer speech perception outcomes in patients with single sided deafness [47]. The categories of etiology used in our analysis do not show any correlation to the AGF slope, leading to the assumption that the etiology is not a factor to be used to predict the AGF slope before implantation. This subsequently means that none of the etiology categories used in the present study can be applied to judge possible beneficial effects of a cochlear implantation for the individual patient. This may be true in general or due to the patient cohort. Maybe the categories were defined too broad, for example, “infection” includes too inhomogeneous etiologies. Additionally, grouping ototoxins and trauma combines two very different causes of deafness with different prognoses for the outcome [28]. It may be that by separating the etiology groups in more precise categories, a correlation to the AGF slope could be detected. Future studies addressing this should be conducted. Another reason may be the measurement of ECAPs, which may need to be advanced, with the ART-measurement not being the optimal stimulation paradigm. In this retrospective study, we used ECAP measurements from the clinical routine. They have been recorded with the aim of proving a functional electrode–nerve interface. Other studies used different inter-phase-intervals to explore neuronal health in animals [10,37]. Such elaborated paradigms may be useful also in human CI users to to allow more detailed insights into the neuronal population of the inner ear [48] and therefore a more effective correlation to be used to predict the outcome of cochlear implantation.

## 5. Conclusions

The ECAP AGF slope is steeper in the apical region in comparison to the basal region of the cochlea. This finding suggests that in the more apical region, there are more and/or healthier neurons than in the basal part. Whether this has an impact on the CI outcome has to be investigated in future studies.

The etiology categories used in this study do not correlate with the AGF slopes measured and can therefore not be used for prediction. The age at implantation and duration of deafness are negatively related to the AGF slope and are therefore perfectly suitable to be used for prediction of the benefit the patient may have by cochlear implantation, since a prediction about the neuronal health status is possible. By this, our study supports the conviction of the CI community that an early implantation is crucial for its success. The consequence should be that patients should be cochlear implanted as early in life and as soon as possible after hearing loss.

## Figures and Tables

**Table 1:** The ECAP AGF slopes of grouped electrodes were significantly correlated (marked in bold) to the duration of deafness and the age at implantation for both data sets analyzed. Analysis A = missing values set to 0; Analysis B = missing values excluded.

	Duration of Deafness				Age at Implantation			
	Analysis A		Analysis B		Analysis A		Analysis B	
electrodes	*p*-value	r^2^	*p*-value	r^2^	*p*-value	r^2^	*p*-value	r^2^
1–12	**0.017**	0.041	**0.032**	0.035	**<0.001**	0.119	**<0.001**	0.134
Grouped electrodes								
1–3 (apical)	0.115	0.018	**0.038**	0.035	**0.020**	0.039	**0.018**	0.045
4–9 (middle)	**0.013**	0.044	**0.014**	0.047	**<0.001**	0.136	**<0.001**	0.156
10–12 (basal)	**0.008**	0.051	**0.012**	0.053	**<0.001**	0.195	**<0.001**	0.287

**Table 2:** Dependence of the mean ECAP AGF slope of all electrodes in data set B on etiology: there was no significant correlation detectable. However, there are reduced effect estimates for all predictors (regression coefficient B).

	Regression Coefficient B	Std.-Error	Beta	Significance
Constant	42.356	7.034		<0.001
Not documented	1.207	7.671	0.029	0.875
Acute hearing loss	−5.328	7.639	−0.130	0.487
Infection	−15.441	8.851	−0.238	0.084
Syndromal/hereditary	−9.018	8.725	−0.144	0.303
Trauma/ototoxins	−8.078	8.122	−0.158	0.322

**Table 3:** Regression coefficient for the ECAP AGF slope of the grouped electrodes and the etiology of hearing loss for both data sets (analysis A and B).

	Analysis A			Analysis B		
	Regression coefficient B			Regression coefficient B		
Electrodes	1–3	4–9	10–12	1–3	4–9	10–12
Constant	64.095	27.714	17.095	64.095	32.333	26.900
Not documented	−10.104	8.390	5.355	−0.186	8.121	2.314
Acute hearing loss	−16.111	1.333	1.341	−6.456	1.932	−2.817
Infection	−27.540	−5.700	−3.262	−24.220	−7.481	−10.233
Syndromal/hereditary	−30.806	−2.803	−4.651	−21.845	0.217	0.100
Trauma/ototoxins	−24.776	0.206	0.383	−17.127	−0.459	−3.855

**Table 4:** Regression coefficient for the ECAP AGF slope of the grouped electrodes and the etiology grouped by occurrence of hearing loss for both data sets (analysis A and B).

	Analysis A				Analysis B			
	Regression coefficient B				Regression coefficient B			
Electrodes	all	1–3	4–9	10–12	all	1–3	4–9	10–12
Constant	27.132	41.641	26.350	14.188	32.246	42.987	28.624	18.372
Not documented	10.344	12.949	10.021	8.385	8.444	13.581	9.098	7.393
Acute	2.569	2.972	1.613	4.079	1.053	4.093	1.807	2.155
Progressive	1.724	2.207	0.362	3.963	−0.335	2.073	−0.760	0.689

**Table 5:** Level of significance for all significant results of correlating the electrodes and the frequency specific residual hearing for data set A and data set B.

	Analysis A (Implant Model 20 mm)		Analyse B (IMPLANTATMODEL 24 mm)
Electrodes	1–3	2	3
Residual hearing			
125 Hz	/	0.035	/
250 Hz	/	0.030	0.041
500 Hz	0.037	0.034	/

**Table 6:** Multivariate analysis of significant variables for the mean ECAP AGF slopes (µV/nC) of all electrodes and of the grouped electrodes 4–9 and 10–12 for both data sets analyzed (A and B). Significant correlations of ECAP AGF slopes and the duration of deafness or the age at implantation are marked in bold.

Multivariate Analysis of Significant Variables for the Mean ECAP AGF slope of All Electrodes						
	Analysis A			Analysis B		
	Regression coefficient B	Std. Error	Sig.	Regression coefficient B	Std. Error	Sig.
Constant	49.805	4.330	<0.001	54.899	4.039	<0.001
Duration of deafness	−0.183	0.097	0.060	−0.148	0.091	0.105
Age at implantation	−0.291	0.073	**<0.001**	−0.282	0.068	**<0.001**
Multivariate analysis of significant variables for the mean ECAP AGF slope of **electrodes 1–3**						
	Analysis A			Analysis B		
	Regression coefficient B	Std. Error	Sig.	Regression coefficient B	Std. Error	Sig.
Constant	/	/	/	73.482	7.318	<0.001
Duration of deafness	/	/	/	−0.298	0.167	0.077
Age at implantation	/	/	/	−0.264	0.124	**0.036**
Multivariate analysis of significant variables for the mean ECAP AGF slope of **electrodes 4–9**						
	Analysis A			Analysis B		
	Regression coefficient B	Std. Error	Sig.	Regression coefficient B	Std. Error	Sig.
Constant	49.522	4.252	<0.001	53.610	3.932	<0.001
Duration of deafness	−0.188	0.095	**0.049**	−0.185	0.089	**0.039**
Age at implantation	−0.309	0.071	**<0.001**	−0.304	0.066	**<0.001**
Multivariate analysis of significant variables for the mean ECAP AGF slope of **electrodes 10–12**						
	Analysis A			Analysis B		
	Regression coefficient B	Std. Error	Sig.	Regression coefficient B	Std. Error	Sig.
Constant	34.933	2.917	<0.001	43.275	2.721	<0.001
Duration of deafness	−0.137	0.065	**0.037**	−0.143	0.065	**0.029**
Age at implantation	−0.266	0.049	**<0.001**	−0.305	0.046	**<0.001**

**Table 7:** Average values of the ECAP AGF slopes (µV/nC) for the different electrode array lengths (mm) for all electrodes and grouped electrodes of both data sets analyzed.

		Electrode Array Length (mm)				
		16 (N = 2)	20 (N = 13)	24 (N = 14)	28 (N = 59)	31.5 (N = 51)
	electrodes	mean ECAP AGF slope				
Data set A	1–12	20.75	24.15	32.40	35.17	28.20
	1–3	44.33	32.77	45.86	52.45	43.43
	4–9	16.33	25.71	31.87	33.45	26.50
	10–12	6.0	12.41	20.02	21.32	16.37
Data set B	1–12	32.94	28.53	41.98	39.37	36.53
	1–3	44.33	35.81	57.74	59.51	54.09
	4–9	25.33	27.32	39.01	36.67	32.93
	10–12	12.00	21.20	26.08	26.03	24.85

## Data Availability

The data presented in this study are available on request from the corresponding author.
